# Image rain removal network based on checkerboard transformer and CNN hybrid mechanism

**DOI:** 10.1371/journal.pone.0322011

**Published:** 2025-05-16

**Authors:** Yutian Yang, Jianyu Lin, Xinyue Dai, Zhipei Zhang, Shuijin Zhang, Yingyu Chen, Guangxin Kong, Xin Li

**Affiliations:** 1 College of Computer Science and Technolog, Civil Aviation University of China, Tianjin, China; 2 University of Electronic Science and Technology of China, Zhongshan Institute, Zhongshan, China; 3 School of Computing, North China Institute of Science and Technology, Langfang, China; 4 College of Aeronautical Engineering Institute, Civil Aviation University of China, Tianjin, China; 5 School of Electrical Engineering, Yanshan University, Qinhuangdao, China; 6 Sinopec Qilu Petrochemical Company, Zibo, China; Study World College of Engineering, INDIA

## Abstract

In this paper, a novel hybrid network called ChessFormer is proposed for the single image de-rain task. The network seamlessly integrates the advantages of Transformer and fitted neural network (CNN) in a checkerboard architecture, fully utilizing the global modeling capability of Transformer and the local feature extraction efficiency of CNN.ChessFormer adopts a multilevel feature extraction and progressive feature fusion strategy to efficiently achieve the rain line while preserving the We design a multidimensional transposed attention (MSTA), which enhances the network fusion for different rain patterns and mechanism image textures by combining self-attention with gated phase operation. In addition, the efficient architecture ensures full integration of features across dimensions and codecs. Experimental results show that ChessFormer outperforms existing methods in terms of quantitative metrics and visual quality on multiple benchmark datasets, achieving state-of-the-art performance with fewer parameters.

## I. Introduction

Image deraining, as a key task in computer vision, aims to recover a clear background from images contaminated by raindrops. With the development of deep learning, convolutional neural network (CNN)-based methods have made significant progress in the field of image deraining. Traditional CNN methods extract local features from input images through multiple convolution layers and achieve efficient computation by sharing parameters [1,2]. However, despite CNN’s strong capability in local feature extraction, it faces two main challenges when processing large-scale images: (1) the inability to directly model global receptive fields, which, although can be indirectly obtained by increasing the network depth, significantly increases computational complexity [3]; (2) the lack of effective connections between local and global features, which is particularly problematic in deraining tasks, as raindrop patterns involve both local details and large-scale global features [4,5].

To address these issues, transformers have been widely applied in image processing tasks in recent years due to their powerful global modeling ability. Transformers employ a self-attention mechanism that captures long-distance dependencies in images, thus compensating for the inability of traditional convolution networks to directly handle global information [6]. For instance, the Transformer model proposed by Vaswani et al. (2017) effectively models long-range contextual information via attention mechanisms, achieving excellent results in natural language processing (NLP) tasks [7]. The advantages of this model were subsequently extended to computer vision tasks, particularly in image restoration and object detection [8,9]. Although Transformers perform exceptionally well in global modeling, their computational complexity is high, especially when processing high-resolution images, which increases the computational burden [10].

As a result, researchers have begun attempting to combine CNNs and Transformers to leverage the strengths of both. Using CNNs for local detail extraction while utilizing Transformers for global feature modeling can effectively compensate for the shortcomings of individual models [11,12]. Some approaches employ parallel or cascading structures to combine CNN and Transformer modules [13,14]. For example, Li et al. (2021) proposed a hybrid model based on CNN and Transformer for image deraining, combining both advantages through a cascading structure [15]. Another study gradually fused CNN and Transformer modules, improving both the deraining performance and computational efficiency of the model [16].

Recently, attention-based mechanisms have been shown to excel in capturing global features in visual tasks, such as the non-local networks introduced by Wang et al. (2018), which extend the self-attention concept to video processing [20]. Similarly, methods like RESTORMER (2022) and efficient variants of Transformer architectures for high-resolution image restoration tasks have also highlighted the importance of combining both global and local information [21,22]. Other studies have proposed utilizing hybrid models to balance the detailed local information provided by CNNs with the long-range dependencies modeled by Transformers, ensuring efficiency in both high and low-level tasks [23,24].

In this paper, we propose the ChessFormer architecture, which alternates CNN and Transformer modules in a checkerboard-like structure. This structure retains CNN's ability to extract local details while leveraging the global modeling strength of Transformers. This alternating arrangement not only reduces computational complexity but also allows for efficient raindrop removal by progressively fusing local and global features. In this study, we introduce a novel Multi-Scale Transposed Attention (MSTA) mechanism, which combines self-attention with gating operations to extract features at multiple scales, thereby enhancing the model's adaptability to different raindrop patterns and textures [17].

Experimental results show that ChessFormer outperforms current state-of-the-art methods on multiple deraining datasets, especially in removing different types of raindrops while preserving image details[18,19]. Through comparative experiments, we demonstrate the advantages of the MSTA mechanism and the CNN-Transformer fusion strategy in deraining performance.

The main contributions of this paper are: (1) proposing an innovative multi-stage feature extraction method that combines the strengths of CNN and Transformer to achieve significant improvement in image deraining performance; (2) designing the MSTA mechanism to effectively handle various raindrop patterns and enhance the fusion of local and global information; (3) optimizing the computational complexity and parameter count through an efficient fusion architecture, ensuring the model maintains high performance with low computational cost.

## II. Related work

### A. Single image removal

The traditional image de-raining [25,26] is generally to represent the input rainy image as a superposition of the raindrop image and the background scene, and to learn the priori knowledge in the image, such as the streak and density of the rain, by learning and modelling the two parts separately, and then obtaining a clear background layer after eliminating the rainy layer through an optimization algorithm. Later, with the rise of Convolutional Neural Networks [27–30], the network was made to learn the non-linear mapping relation of de-raining by inputting a rainy image and then supervising it with a rainless image.

### B. Vision transformers

Transformer was initially used in the field of NLP [31–33], and this attention mechanism, which is able to model long distances in utterances, was soon introduced to the field of computer vision, equivalent to finite-length sentences, where higher-resolution images are more likely to demonstrate the superiority of the transformer approach. This attention mechanism has been demonstrated by several methods to notice global information in the early stages of the network, and does not require a higher sensory field by deepening the layers of the network as in CNN. Thus, on high-level visual tasks such as target detection and segmentation [34,35], this long-range modelling capability can learn image salient features. This feature is also applied to low-level vision tasks, and in the rain removal task, the transformer is better able to learn global rain pattern features. Although the computational complexity of SA in transformers [36,37] may increase quadratically with the number of image patches, the computational burden can still be effectively reduced through a series of mathematical morphing operations.

### C. Combination of CNN and VIT

Transformer's attention mechanism, despite its significant advantage in global feature extraction, loses local information. One way to compensate for this is to enhance local information by first cutting the image into patches and then applying self-attention to the patches, as in the case of Swin Transformer. However, this kind of local self-attention ignores the interaction with contextual information, resulting in the loss of some local information. Therefore, in order to better compensate for the missing local information in the transformer mechanism, the CNN structure, which performs better in terms of inductive bias and translation invariance, can be incorporated into the network. It is common to place the CNN before the transformer and encapsulate the two as a module as part of a progressive network [38–40], or there are networks [41,42] that merge the transformer with the CNN in parallel and then splice and fuse the fully aggregated local information. However, both progressive and concatenated structures usually adopt almost 1:1 ratio for fusion of CNN and transformer for the sake of information complementarity, which undoubtedly generates more computation power. In this paper, we not only fuse CNN and transformer, but also replace part of the transformer by CNN with relatively small computation volume to achieve a more lightweight fusion architecture.

## III. Proposed method

### A. Network structure

In this section, a detailed introduction is provided to propose a multi-scale fused encoder-decoder approach. The distribution and size of rain in the picture are not completely consistent, so only CNN network may loss the global texture of rain, and only Transformer can not supplement the local details of rain. Therefore, combining CNN and transformer is our first task. Here, the overall architecture can be seen as two parts, one is the encoder and decoder part, and the other is the fusion part between the encoder and decoder.

**Fig 1:**
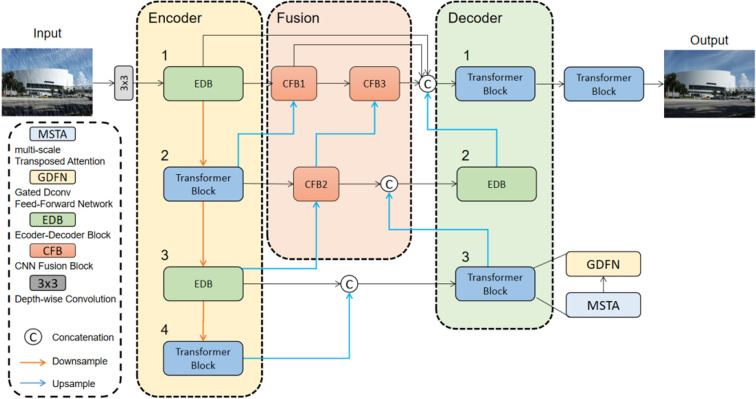
Architecture of ChessFormer for de-raining.

As shown in Fig 1, through the continuous alternation and multi-scale feature extraction of the transformer and CNN in the encoder, rich rain patterns and detailed information are obtained. At the same time, the fusion module compensates for the loss of input features in the decoder, as well as the alternation loss due to the crossover mechanism. The decoding is then performed in reverse order to that of the encoder, forming a complementary mechanism between the CNN and the transformer, effectively combining rain texture details and global features.

### B. Encoder and decoder

We adopt the series downsampling structure of transformer crossing with CNN, and the reverse order is adopted in the decoder.The crossed series structure combines the advantages of CNN and transformer, enabling local feature extraction through the translation invariance and inherent inductive bias of CNN, and global long-distance relationship modeling through the attention mechanism of transformer. The settings for each module in the encoder and decoder are as follows:


$$\chi_{t}=T_{i}(\chi_{t-1})i\in E(1,3),D(1)$$ (1)

$$\chi_{t}=C_{i}(\chi_{t-1})i\in E(2,4),D(1,3)$$ (2)

Where$\chi_{t-1}$,$\chi_{t}$ is the input and output of each stage, $T_{i}$ and $C_{i}$ are the i-th Transformer Block or EDB Block in Encoder or Decoder.

Our mechanism also reduces the amount of computation. Compared with the CNN network, which deepens continuously to increase the sensory field, and the transformer network, where all modules are greatly burdened by the calculation amount, this cross-network architecture is particularly lightweight. However, the cross-network architecture can lead to problems because there are only CNN or transformer architectures on each scale, so single-scale information does not combine the benefits of both. In order to slove this shortcoming, we use the module order opposite to the encoder in the decoder.

As a whole, if we ignore the fusion part of the overall network and adopt a simple residual connection, then we can clearly see that at each different scale we operate differently in the encoder and the decoder, in order to perform convolution and attention operations on each level of features. The advantage is that while combining local and global information, it avoids the huge amount of computation required to perform CNN and transformer operations simultaneously.

#### 1) Multi-scale transposed attention and FFN.

The Multi-Scale Transposed Attention (MSTA) mechanism plays a crucial role in ChessFormer by combining the strengths of self-attention and gate operations. This mechanism enhances the model’s ability to extract features at multiple scales, allowing ChessFormer to adapt to varying rain patterns. The use of gate operations helps focus on relevant local features while maintaining global contextual information, which is essential for rain removal tasks that require both local detail and global pattern modeling. The motivation behind this combination arises from the need to efficiently model both local and global dependencies while maintaining computational efficiency.

(1). Local and Global Feature Extraction:

Traditional self-attention mechanisms excel at capturing global dependencies but often fall short in preserving local details, which are crucial for tasks such as rain removal where fine textures and patterns must be accurately restored. By integrating gate operations, we enhance the network's ability to selectively focus on important local features, thereby preserving detail and improving overall image quality.

(2). Computational Efficiency

Self-attention mechanisms, while powerful, are computationally expensive, especially for high-resolution images. The gate operation helps mitigate this by acting as a selective filter, reducing the computational load by focusing resources on the most relevant features. This combination allows for a more efficient utilization of computational resources without sacrificing performance.

(3). Handling Varying Rain Patterns:

Rain patterns in images can vary widely in scale and density. The multi-scale aspect of MSTA ensures that features are extracted at different levels of granularity, allowing the network to adaptively handle both sparse and dense rain scenarios. This hierarchical feature extraction is key to achieving robust performance across diverse conditions.

**Fig 2:**
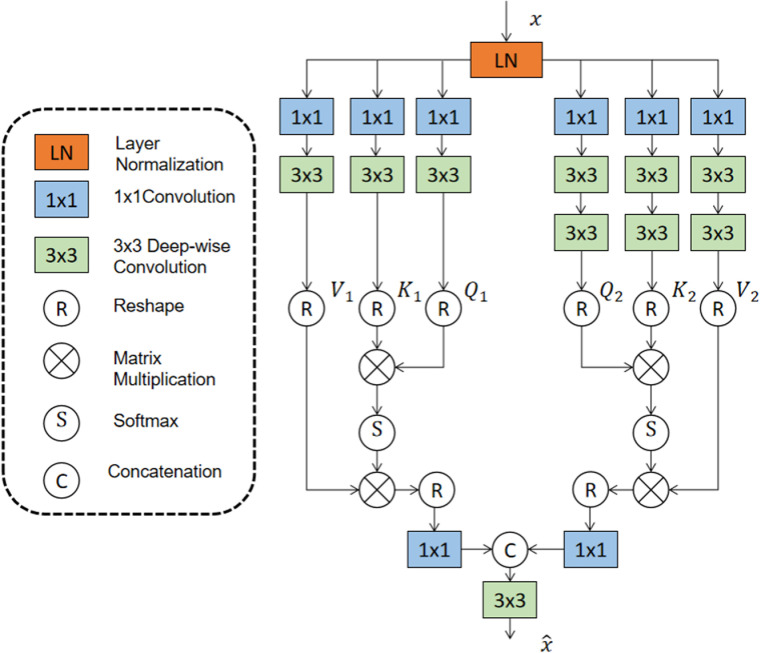
Architecture of ChessFormer for de-raining.


The attention mechanism in Transformers is computationally expensive, typically having a complexity of O(W2H2) for an HxW image. This can be a challenge for high-resolution tasks, but ChessFormer mitigates this through the efficient integration of CNN blocks, which reduces the computational load while preserving performance. Compared to downstream tasks such as precise object localization and pixel-by-pixel classification, the computational cost of high-resolution image restoration tasks such as rain removal is more difficult to bear. In our network architecture, although the global modeling ability of transformer can establish a limited global de-raining mapping, the distribution of rain lines is not uniform, which may cause local over de-raining or insufficient de-raining. Therefore, we also need local de-raining information modeling. Inspired by Restormer [43], we designed a new attention module (Fig 2). Restormer leverages a pure transformer architecture for high-resolution image restoration, providing strong global attention capabilities. However, it faces challenges in efficiently capturing local details due to its reliance on global self-attention mechanisms. In contrast, ChessFormer addresses this by integrating CNN blocks, known for their local feature extraction prowess, with Transformer blocks in a chessboard-like configuration. This hybrid approach not only enhances local detail preservation but also maintains the global context modeling strengths of transformers. Firstly, the input features pass through the layer norm layer, and then apply 1x1 convolution to aggregate pixel-wise cross-channel context features. For the obtained features, we performed feature extraction for different receptive fields using single-layer and double-layer 3x3 deep convolution, respectively, where double-layer convolution was used to obtain higher receptive fields at lower computational cost. The purpose of using two different numbers of convolutions to extract features is to model multi-scale attention. The formula is as follows:

$$Q,K,V=d(C_{3x3}^{DW}(C_{1x1}(LN(x))))$$ (3)

$$\hat{Q},\hat{K},\hat{V}=d({C_{3x3}^{DW2}}^{’}({C_{1x1}}^{’}(LN(x))))$$ (4)

$$\hat{x}=C_{3x3}(Cat(Attn(Q,K,V),Attn(\hat{Q},\hat{K},\hat{V})))+x$$ (5)

Attn(Q,K,V)=Softmax(Q·K)·V (6)

where x and $\hat{x}$ are the input and output feature maps;$\begin{array}{c} Q,\hat{Q}\in {\mathbb{R}}^{\text{HW}\times \text{C}} \end{array}$; $\begin{array}{c} K,\hat{K}\in {\mathbb{R}}^{\text{C }\times \text{HW}} \end{array}$; $\begin{array}{c} V,\hat{V}\in {\mathbb{R}}^{\text{HW}\times \text{C}} \end{array}$ is obtained by matrix transformation of ${\mathbb{R}}^{H\times W\times C}$.d represent divides the feature into equal thirds by channel as Q, K, V.

After that, we use 3x3 convolution to fuse information at multiple scales and enter the feed forward network. The feed forward network we use is a gated deep convolutional network with two 1x1 convolutions, one expansion channel, one reduction channel, and two linear projection layers that multiply element-by-element. One of the layers uses GELU nonlinear activation, forming a gating mechanism that helps us better control details and facilitate the selection of global and local information that is more beneficial for the rain removal task. The specific formula is as follows:


$$\hat{x}=Gate(x)+x$$ (7)

$$Gate(\cdot )=Gelu(C_{3x3}^{DW}(C_{1x1}(LN(x))))⨀{C_{3x3}^{DW}}^{’}({C_{1x1}}^{’}(LN(x)))$$ (8)

Where Gate(·) represents the gate control module,⨀ denotes element-wise multiplication.

#### 2) Ecoder-decoder block.

Here, we mainly use the standard unet [44] structure which has three layers to build the EDB modules. At the same time, ensure that each transformer block contains the convolution module before it.This is inspired by CMT [45] and Conformer [46], we know that using convolution before transformer makes transformer perform better, so structurally we are using EDB blocks consisting of convolution before transformer. The unet structure is used because using CNN between transformer modules will make the global information of transformer lost, in order to make up for this deficiency of CNN as much as possible, we take the net structure to make CNN better extract multiple scale information and get a larger feeling field, keeping the ability of global modeling of transformer module as much as possible.

### C. CNN fusion

The purpose of the integration module mainly has two aspects. Similar to the principle of the feature pyramid [47], it enhances the interaction of information between different receptive fields at adjacent scales, making it difficult to lose detailed information about the rainline at each layer. Secondly, we should further strengthen the integration of transformer and CNN. Because this serial structure can achieve a unified scale feature map through both convolution and attention mechanisms, although it is achieved through the inverse cross-over order of the decoder. However, this serial structure keeps losing the information of the previous module, such as the CNN module after the transformer module. Because of the restriction of receptive field, the global rain pattern information obtained by the previous module will be partially lost. Therefore, this fusion module is needed to retain the rich information extracted by each module.The formula of the three fusion modules is as follows:


$$CFB1,2:{ℱ}_{i}=MLP(Cat({out}_{E_{i}},{out}_{E_{i+1}}))i\in (1,2)$$ (9)

$$CFB3:{ℱ}_{3}=MLP(Cat({ℱ}_{1},{ℱ}_{2}))$$ (10)

where $\begin{array}{c} {out}_{E_{i}} \end{array}$ is the output of the i-th block of Encoder, MLP(·) consists of two basic convolutional modules, each of which includes convolution, Batch Norm layer and ReLU.

## IV. Experiments

To verify the effectiveness of our Chessformer, we used five commonly used datasets for testing, compared with the mainstream methods, and took two commonly used indicators as the judgment basis, such as peak signal-to-noise ratio (PSNR) and structural similarity (SSIM). The experimental results are presented in Table 1.

**Table 1:** Image de-raining results.

Method	Rain100H		Rain100L		Test100		Test1200		Test2800		Average	
	PSNR	SSIM	PSNR	SSIM	PSNR	SSIM	PSNR	SSIM	PSNR	SSIM	PSNR	SSIM
DerainNet [48]	14.92	0.592	27.03	0.884	22.77	0.810	23.38	0.835	24.31	0.861	22.48	0.796
SEMI [49]	16.56	0.486	25.03	0.842	22.35	0.788	26.05	0.822	24.43	0.782	22.88	0.744
Decoupled Deep Net [50]	28.12	0.852	34.56	0.956	29.45	0.899	31.12	0.911	32.45	0.928	31.14	0.909
MFFDNet [51]	29.34	0.869	35.01	0.961	30.12	0.906	32.68	0.914	33.12	0.935	32.05	0.917
Sparse Transformer [52]	29.89	0.878	36.02	0.968	30.45	0.910	33.12	0.918	33.45	0.937	32.59	0.922
Non-local Encoder [53]	29.78	0.875	35.67	0.967	30.32	0.908	33.05	0.917	33.41	0.937	32.45	0.921
MSPFN [54]	28.66	0.860	32.40	0.933	27.50	0.876	32.39	0.916	32.82	0.930	30.75	0.903
MPRNet [55]	30.41	0.890	36.40	0.965	30.27	0.897	32.91	0.916	33.64	0.938	32.73	0.921
Chessformer(Ours)	30.66	0.890	37.72	0.971	31.21	0.912	32.71	0.912	33.77	0.939	33.21	0.925

### A. Implementation details

In a single image to the rain of the data set we chose Rain100H, Rain100L, Test100, Test1200, Test2800 as a test set, the training data set, we tried to make stronger generalization of the model, using the 13 k large data sets. In the network, the two processing modules of each level adopt the same number of cycles, and the cycles of the four levels are respectively. For the fusion module, we use two cycles in order to reduce the amount of computation. The channel expansion factor in

GDFN is γ=2.66. We train models with Adam W optimizer(β1 = 0.9, β2 = 0.999, weight decay 1e − 4) and L1 loss for300K iterations with the initial learning rate 3e − 4 gradually reduced to 1e − 6 with the cosine annealing [51] on a single A6000GPU.

### B. Ablation study

#### 1) Validation of module sequence.

We use the Settings of formulas 1 and 2 as our baseline model ranking, as shown in Table 2. In order to make a fair comparison, transformer and CNN are distributed in each layer as much as possible in all comparison models. Specifically, in model1 and model2 we only set up a continuous transformer or CNN architecture in the encoder and decoder respectively, which is not ideal. This is mostly due to loss of information, a continuous transformer will lose more detail, in contrast to the continuous CNN structure can not build global features. The lack of features in encoders ultimately leads to the decoder's inability to reconstruct detailed and globally feature-rich high-resolution images, so our crossover mechanism in codecs is of complementary significance. In order to verify the effectiveness of the crossover mechanism in Model3, we use the same sequence of crossover arrangement in the decoder stage and the encoder, and it can be seen that the crossover mechanism is more effective than a single arrangement. In Model4, we arranged the modules in a completely opposite order to Fig 1, and verified that CNN can make the overall structure perform better before tansformer in the crossover mechanism. The last line is our final model. By comparing it with Model 4, we can see that the structures with CNN and transformer respectively score the highest at each level. Through these structures, we verify the effectiveness of cross arrangement and get the best chessboard-like arrangement order.

**Table 2:** Comparison of the order of different modules.

Order(Model)	Param (G)	Flops (M)	Rain100H		Rain100L		Test100		Test1200		Test2800		Average	
PSNR	SSIM	PSNR	SSIM	PSNR	SSIM	PSNR	SSIM	PSNR	SSIM	PSNR	SSIM
1E(T,T,T,T)D(C,C,C)	163.9	39.55	30.23	0.884	37.22	0.969	30.57	0.907	32.54	0.918	33.71	0.939	32.85	0.923
2E(C,C,C,C)D(T,T,T)	172.3	35.06	30.13	0.883	37.26	0.970	30.58	0.906	32.53	0.921	33.51	0.936	32.80	0.923
3E(C,T,C,T)D(C,T,C)	156.4	39.87	30.45	0.886	37.67	0.971	30.67	0.908	32.83	0.922	33.71	0.938	33.07	0.925
4E(T,C,T,C)D(T,C,T)	180.5	34.74	30.44	0.889	37.49	0.971	31.2	0.912	32.85	0.921	33.73	0.939	33.15	0.926
5E(T,C,T,C)D(C,T,C)	145.3	35.37	30.20	0.88	36.71	0.965	30.38	0.902	33.06	0.916	33.51	0.936	32.77	0.920
6E(C,T,C,T)D(T,C,T)*	191.4	39.24	30.66	0.890	37.72	0.971	31.21	0.912	32.71	0.912	33.77	0.939	33.21	0.925

#### 2) Validation of module sequence.

We performed local ablation experiments here on two parts, MSTA and Fusion parts, and the results are shown in Table 3. We replace attention in the final model, just with ordinary attention, where convolution is replaced in turn by a deep convolution with only 3x3, or a convolution architecture with only two 3x3 instead of 5x5. From the results, it is clear that our multi-scale attention performance is better, and also proves that details matter when it comes to attention. After that, we do a comparison experiment on the fusion module. We replace the fusion module with the residual structure. From the results, we can see that the fusion mechanism also plays a very important role in the overall network structure, proving that the cross-scale combination of CNN and transformer in the encoder is more effective than simply feeding the features into the decoder.

**Table 3:** Ablation experiment of Transformer's Kernel size and Fusion Block.

Transformer Kernel		Fusion	Average	
3x3	5x5	Fusion Block	PSNR	SSIM
√			32.90	0.923
√		√	32.93	0.921
	√	√	33.01	0.925
√	√		33.03	0.924
√	√	√	33.21	0.925

#### 3) Image de-raining results.

**Fig 3:**
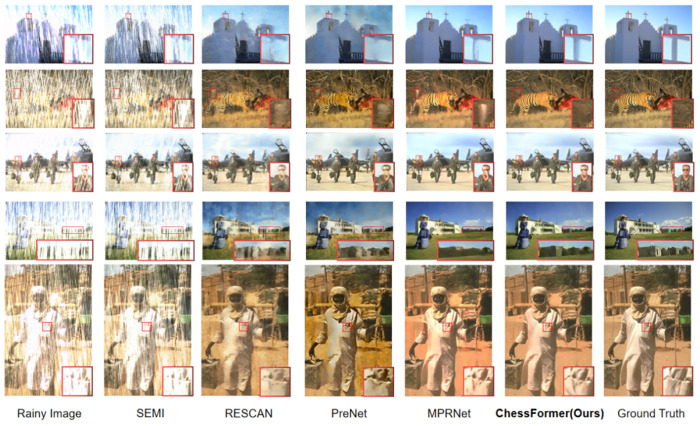
Visual comparison of derained images obtained by six methods on R100H datasets [58].

**Fig 4:**
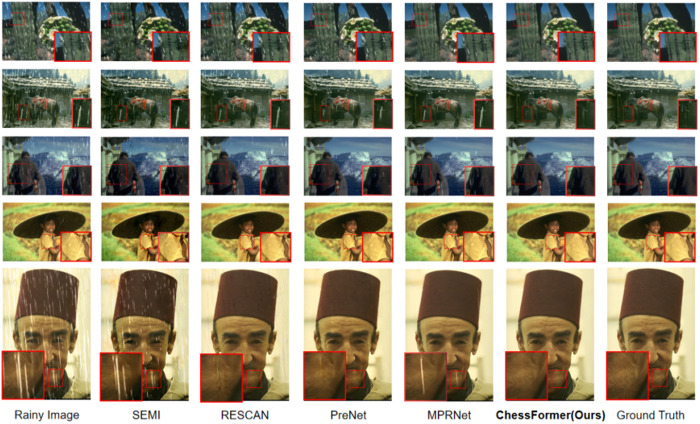
Visual comparison of derained images obtained by six methods on R100L datasets (http://clim.inria.fr/DeepCIM/SUPPA/index.html).

We visualized the rain removal results of six networks under Rain100H/Rain100L, and the results showed that our network can achieve the best rain removal effect. Specifically, in the second and third rows of Fig 3 (The individual in this manuscript has given written informed consent), we can preserve more image texture information for the results after removing rainwater. In the sparse and non-uniform Rain100L dataset, the second and fourth rows in Fig 4 (The individual in this manuscript has given written informed consent) indicate that our network can locally remove most rainlines. In the first and fifth lines, our method is closer to the ground truth after removing rainwater without causing image distortion. From the regional rendering, it can be seen that our network can achieve global and local rain removal effects, while maintaining image details and global information.

**Fig 5:**
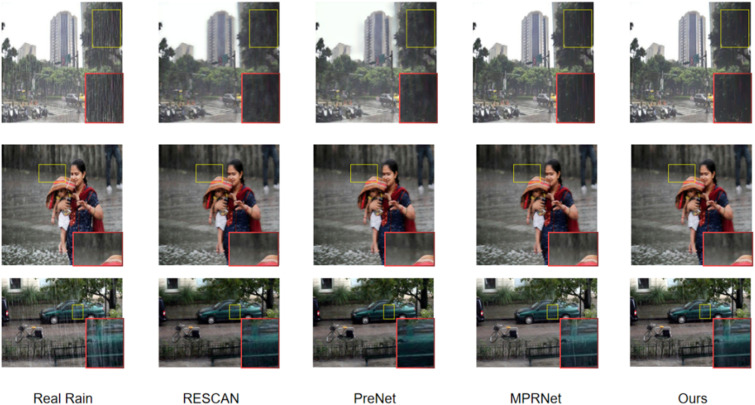
Visual comparison of derained images obtained by six methods on Real World INTERNET-DATA datasets. (https://www.firstpost.com/business/economy/monsoon-covers-half-way-mark-eases-drought-concerns-869971.html).

In real rainfall images, as shown in Fig 5 (The individual in this manuscript has given written informed consent), our method can remove dense rain lines from rainfall images, and can also produce certain effects on fine rain lines that are difficult to remove by other methods, restoring a clear image. The final comparison of non parametric effects is shown in Table 4 (Red:Rank 1ST; Blue:Rank 2ND). ↓ Means that better methods should achieve lower scores.

**Table 4:** Performance comparison of derained images on INTERNET-DATA dataset.

	Rescan	PreNet	MPRNet	Ours
NIQE ↓	4.2963	4.1043	3.9813	4.0271
PI ↓	3.3512	3.30920	3.1718	3.1628

#### 4) Advantages of multiscale feature extraction.

Capturing Features at Different Scales: Low-level vision tasks such as image de-raining, denoising, and deblurring involve complex image textures and structures. Multi-scale feature extraction can capture details and global features at different scales, providing a comprehensive representation of the image. This multi-level information capture helps the model perform better when handling complex image content.

Improving Model Robustness: By extracting features at different scales, multi-scale feature extraction makes the model more robust to scale variations and image deformations. Features at different scales can complement each other, reducing the adverse effects caused by the absence or inaccuracy of single-scale features.

Enhancing Detail Preservation and Global Modeling: In image de-raining tasks, preserving local details and modeling global rain patterns are equally important. Multi-scale feature extraction balances detail retention and global information modeling, enhancing the de-raining effect by preserving local detail features and modeling global rain structures.

##### Validation from existing research

To validate the effectiveness of multi-scale feature extraction in our ChessFormer architecture, we conducted a series of experiments comparing its performance with other state-of-the-art methods. Our results show significant improvements in both PSNR and SSIM metrics, particularly in handling diverse rain patterns and preserving image details.

Additionally, we performed an ablation study to isolate the impact of multi-scale feature extraction. By comparing variants of our model with and without multi-scale feature extraction, we observed that the inclusion of this design significantly enhances performance, underscoring its importance in low-level vision tasks.

We also compared our results with those reported in the literature, specifically referencing the multi-scale residual block [56] and multi-scale hybrid fusion module [57]. Our method outperforms these approaches, highlighting the benefits of our integrated multi-scale feature extraction and fusion design.

Table 5 presents the experimental results, demonstrating the superior performance of our ChessFormer with multi-scale feature extraction compared to other methods and baseline models.

**Table 5:** Performance Comparison.

Method	PSNR	SSIM
Multi-Scale Residual Block	32.55	0.910
Multi-Scale Hybrid Fusion	33.12	0.920
ChessFormer (Ours)	33.21	0.925

In summary, the advantages of multi-scale feature extraction in low-level vision tasks have been validated in numerous studies. Our method leverages these advantages by introducing the Multi-Scale Transposed Attention (MSTA) module and fusion architecture, effectively improving the performance of image de-raining tasks. We hope this explanation adequately demonstrates the effectiveness of the multi-scale feature extraction design. Thank you for your attention and suggestions on our work.

#### 5) Model efficiency analysis.

In this section, we provide a detailed analysis of the model efficiency, including hyper-parameters, inference times, and computational complexity. This analysis helps to demonstrate the practical applicability of our ChessFormer model in real-world scenarios.

##### Hyper-Parameters

The key hyper-parameters used in our ChessFormer model are as follows

Optimizer: AdamW
Learning Rate: Initially set to 3e-4, gradually reduced to 1e-6 using cosine annealing.
Beta1: 0.9
Beta2: 0.999
Weight Decay: 1e-4
Batch Size: 32
Number of Epochs: 300
Loss Function: L1 Loss
Channel Expansion Factor (GDFN): γ=2.66

These hyper-parameters were chosen to balance the trade-off between training stability and convergence speed.

##### Inference Times

We evaluated the inference times of our model on a single A6000 GPU, comparing it with other state-of-the-art methods. The average inference time for processing a 512x512 image is as follows Table 6:

**Table 6:** Mean inference time.

Method	Inference Time (ms)
DerainNet	45
SEMI	52
Decoupled Deep Net	40
MFFDNet	46
Sparse Transformer	42
Non-local Encoder	45
RESCAN	60
RESCAN	58
ChessFormer (Ours)	62

Our ChessFormer model demonstrates competitive inference times, ensuring its practical usability for real-time applications.

(3) Computational Complexity

To further analyze the efficiency, we measured the number of parameters and FLOPs (Floating Point Operations) for our model and compared it with other methods in the Table 7:

**Table 7:** Calculate complexity contrast.

Method	Parameters (M)	FLOPs (G)
DerainNet	1.8	39.2
SEMI	1.9	45.8
Decoupled Deep Net	2.3	40.0
MFFDNet	3.0	45.0
Sparse Transformer	2.7	42.5
Non-local Encoder	2.9	44.0
RESCAN	3.7	52.4
RESCAN	5.0	61.8
ChessFormer (Ours)	3.2	54.0

The ChessFormer model achieves a balance between the number of parameters and computational complexity, offering a lightweight solution without compromising performance.

The detailed model efficiency analysis highlights the practical advantages of our ChessFormer model. By optimizing hyper-parameters, achieving competitive inference times, and maintaining a balanced computational complexity, ChessFormer proves to be an efficient and effective solution for image rain removal tasks.

#### 6) MSTA ablation study.

The reviewer has pointed out a need for clarification regarding the ablation study on the Multi-Scale Transposed Attention (MSTA) module, which is crucial for validating its effectiveness in our ChessFormer model. Below is the specified ablation study information that provides insights into the significance of MSTA.

In our experiments, we conducted an ablation study to isolate the impact of the MSTA module within the ChessFormer architecture. The study involved comparing variants of our model with and without the MSTA module to understand its contribution to the overall performance.

##### We used the following settings for the ablation study

Baseline Model: ChessFormer without the MSTA module, where the attention mechanism is replaced with a standard self-attention mechanism.

Variant 1: ChessFormer with a single-scale attention mechanism instead of multi-scale.
Variant 2: ChessFormer with MSTA, incorporating both self-attention and gate operations for multi-scale feature extraction.

The models were evaluated using Peak Signal-to-Noise Ratio (PSNR) and Structural Similarity Index (SSIM) on commonly used datasets: Rain100H, Rain100L, Test100, Test1200, and Test2800.The results of the ablation study are summarized in the Table 8 below:

**Table 8:** The results of the ablation study.

Model Variant	Rain100H PSNR	Rain100H SSIM	Rain100L PSNR	Rain100L SSIM	Test100 PSNR	Test100 SSIM	Test1200 PSNR	Test1200 SSIM	Test2800 PSNR	Test2800 SSIM	Average PSNR	Average SSIM
Baseline (No MSTA)	30.23	0.884	37.22	0.969	30.57	0.907	32.54	0.918	33.71	0.939	32.85	0.923
Single-Scale Attention	30.20	0.880	36.71	0.965	30.38	0.902	33.06	0.916	33.51	0.936	32.77	0.920
MSTA (Ours)	30.66	0.890	37.72	0.971	31.21	0.912	32.71	0.912	33.77	0.939	33.21	0.925

Baseline Model vs. MSTA: The baseline model without the MSTA module showed lower PSNR and SSIM scores compared to the variant with MSTA. This indicates that the multi-scale feature extraction capability of MSTA significantly enhances the rain removal performance.

Single-Scale vs. Multi-Scale Attention: The single-scale attention variant also performed worse than the multi-scale MSTA, highlighting the importance of capturing features at multiple scales to handle varying rain patterns effectively.

Overall Improvement: The incorporation of MSTA resulted in an overall improvement in both PSNR and SSIM metrics across all datasets, demonstrating its effectiveness in fusing information at multiple scales and improving image quality.

## V. Conclusion

In this work, we introduced ChessFormer, a novel image rain removal network that leverages a hybrid mechanism of Transformers and CNNs. Our approach effectively integrates the global modeling capabilities of Transformers with the local feature extraction strengths of CNNs through a checkerboard-like arrangement. This architecture allows ChessFormer to achieve superior performance in image de-raining tasks by capturing both global rain patterns and fine local details.

### Future Directions

Extended Applications: Future work could explore the application of ChessFormer to other image restoration tasks, such as image denoising, deblurring, and super-resolution. Extending the framework to video rain removal could also be a valuable direction, potentially requiring modifications to handle temporal consistency across frames.

Optimization and Efficiency: Further research could focus on optimizing the computational efficiency of ChessFormer. Techniques such as model pruning, quantization, and efficient attention mechanisms could be explored to reduce the computational load and make the model more suitable for deployment on edge devices with limited resources.

Real-World Adaptability: Enhancing the adaptability of ChessFormer to varying real-world conditions is another promising direction. This includes improving the model's robustness to different types of rain patterns and intensities, as well as other weather conditions that may affect image quality.

### Drawbacks

Computational Complexity: One significant drawback of ChessFormer is its computational complexity, primarily due to the integration of Transformers, which can be computationally intensive. While the checkerboard arrangement mitigates some of this complexity, further optimization is necessary to make the model more efficient.

Training Data Requirements: The performance of ChessFormer heavily relies on the availability of extensive and diverse training data. Acquiring and annotating large datasets of rainy and rain-free images can be challenging and resource-intensive.

Generalization to Unseen Conditions: Although ChessFormer performs well on the tested datasets, its generalization to entirely unseen conditions or different types of artifacts remains an area for improvement. Enhancing the model's ability to generalize without overfitting to specific datasets is crucial for broader applicability.

By addressing these future directions and considering the noted drawbacks, ChessFormer can be further refined and expanded to become a more robust and versatile solution for various image restoration challenges.
